# Evaluation of the diagnostic management of deep vein thrombosis in the emergency department of a tertiary hospital in Santa Catarina, Brazil: a cross-sectional study

**DOI:** 10.1590/1677-5449.202002171

**Published:** 2022-10-03

**Authors:** Lucas Tramujas, Márcio Mesquita Judice, Angela Bueno Becker

**Affiliations:** 1 Hospital Governador Celso Ramos, Florianópolis, SC, Brasil.; 2 Instituto de Pesquisa Hcor, São Paulo, SP, Brasil.

**Keywords:** venous thrombosis, diagnosis, clinical protocols

## Abstract

**Background:**

Venous thromboembolism is an entity that encompasses both deep vein thrombosis and pulmonary thromboembolism. Although protocols for the diagnosis of these diseases are well defined, there is evidence of inappropriate use of diagnostic resources.

**Objectives:**

To define the epidemiological profiles of patients admitted to the emergency department with suspected deep vein thrombosis, to determine rates of inappropriate ordering of D-dimer assays and color venous Doppler echocardiography of the lower limbs, and to identify whether these requests followed the recommendations contained in the 2015 Brazilian Society of Angiology and Vascular Surgery guidelines.

**Methods:**

We conducted a cross-sectional observational study that retrospectively evaluated 168 patients with suspected deep vein thrombosis for whom D-dimer assays were requested. The most common risk factors were measured and the pretest probability was calculated with the Wells score. The epidemiological profile of these patients and the rates of inappropriate D-dimer testing were assessed using descriptive statistics.

**Results:**

The D-dimer requests were inadequate in 55 (32.7%) patients. Venous color Doppler ultrasound was used to examine the lower limbs of 14 (8.3%) of the patients with a low probability according to the Wells score and a negative D-dimer result. No additional diagnostic methods were used in 19 (11.3%) of those with a low probability according to the Wells score and a high D-dimer result. There was unnecessary use of CDUS in 35 (20.8%) cases. The overall rate of inappropriate workup was 53.5%.

**Conclusions:**

Differences were found between clinical practice and the recommendations for diagnostic evaluation of patients with suspected deep vein thrombosis, with inappropriate use of diagnostic tests.

## INTRODUCTION

Data from American records show that the incidence of venous thromboembolism (VTE) has remained constant over time, with a rate of 0.7 to 1.4 cases per 1,000 people-years.^1^ It is estimated that around two thirds of these are deep venous thrombosis (DVT) cases.^2^^,^^3^

Clinical diagnosis of DVT is a challenge, since the classic signs and symptoms are not always displayed. Therefore, a combination of clinical findings with risk factors grouped in a prediction system and use of supplementary tests is the best way of making a diagnosis.^4^

Use of tests for D-dimer (DD), a product of degradation of fibrin that originates during the occurrence of thrombotic events, and use of color Doppler ultrasonography (CDUS) of the veins of the lower limbs both play a central role in investigation of suspected DVT cases.^5^^,^^6^

Serum DD levels increase significantly in a series of diseases, with age, and during physiological situations such as pregnancy, making them a marker with high sensitivity but low specificity, and a high negative predictive value for VTE.^7^ The main recommendation is therefore that DD assay should be used as an initial workup test in situations in which there is a low pre-test probability of a diagnosis of DVT, since a negative test result is sufficiently accurate to rule out the possibility of the disease.^8^^,^^9^

However, diagnosis by imaging is necessary to confirm a diagnosis of DVT. In this situation, CDUS is the diagnostic method of choice, with 96% sensitivity and specificity exceeding 98%, depending on the region examined.^10^

In 2012, in the United States, the American Board of Internal Medicine (ABIM) launched a campaign called Choosing Wisely International. This initiative invokes the “less is more” paradigm, urging physicians not to conduct unnecessary tests.^11^

Recent studies have revealed inappropriate use of diagnostic resources for assessment of VTE, particularly so in the few Brazilian studies that have investigated the issue.^12^^-^14

The present study is therefore designed to generate additional data on diagnostic management of DVT, identifying possible mismatches in the investigation of these patients and indirectly highlighting the possible economic impact of inappropriate use of diagnostic resources.

Therefore, the principal objectives of this study were to evaluate the clinical and epidemiological profile of patients with suspected DVT seen at a tertiary hospital in Santa Catarina, Brazil, to calculate the rate of inappropriate requests for DD tests and CDUS examinations, and to determine whether DVT investigations were conducted in accordance with the recommendations set out in the 2015 Brazilian Society of Angiology and Vascular Surgery (SBACV) DVT diagnosis and treatment guidelines.^15^

## METHODS

This was a cross-sectional observational study that analyzed requests for DD tests for patients with suspected DVT at the emergency department of the Hospital Governador Celso Ramos (Florianópolis, state of Santa Catarina, Brazil), from January to December of 2018. Open Epi software was used to perform the sample size calculation.^16^ The estimated sample size was 139 individuals, considering an arbitrary 10% estimated overall frequency of inappropriate diagnostic management, in view of the wide range of rates presented in the literature on patients with suspected DVT.^17^ Absolute precision was 5% and the significance level was set at 5%.

Variables were collected using a research protocol developed by the researchers.

Data provided by the institution’s laboratory were used to identify all patients for whom a DD assay was ordered. Their medical records were then analyzed.

The following inclusion criteria were adopted: (1) age greater than or equal to 18 years; (2) patients with signs and symptoms suggestive of DVT, such as pain, edema, cyanosis, clubbing of calves, dilatation of the superficial vein system, or compatible changes in the limb involved observed during physical examination; and (3) just one DD assay from each patient seen in emergency was included in the analysis.

The exclusion criteria were as follows: (1) clinical variables missing that are needed to calculate probability with the Wells score (WS); (2) suspected DVT in inpatients; and (3) patients who had been seen by the research investigators in the course of their clinical care work.

Major risk factors for DVT were recorded, as follows: age greater than 65 years; obesity (body mass index [BMI] greater than or equal to 30 kg/m^2^); cancer diagnosis during the previous 6 months; chronic venous disease; prior DVT; orthopedic procedures such as hip or knee joint replacement or knee arthroscopy; immobility, defined as suppression of joint movements, whether because of neurological or musculoskeletal reasons or because of a surgical procedure; trauma during the previous month; use of oral contraceptives or hormone replacement therapy; pregnancy; puerperium; genetic or hereditary thrombophilias; and family history of VTE.^15^

The tool used to calculate the pre-test probability of DVT was the modified WS, for which scores of 3 or more points are defined as a high probability of DVT, scores from 1 to 2 points as a moderate probability, and scores from 0 to -2 points as a low probability (Table 1).^6^^,^^15^^,^^18^

**Table 1:** Modified Wells score for deep venous thrombosis (DVT), used to assess patients with suspected DVT seen at a tertiary hospital in Santa Catarina, Brazil, from January to December of 2018.

**Clinical findings**	**Points**
Active cancer (patient has had treatment for cancer within previous 6 months or is currently receiving palliative treatment)	1
Paralysis, paresis, or immobilization of lower extremity	1
Bedridden for 3 days or more or major surgery within last 4 weeks	1
Increased sensitivity along the path of veins of the deep vein system	1
Edema involving entire limb	1
Edema of the calf > 3 cm in comparison with asymptomatic side (measured 10 cm below the tuberosity of the tibia)	1
Pitting edema in involved leg (unilateral)	1
Collateral superficial veins	1
Prior documented DVT	1
Alternative diagnosis more likely than DVT	-2

Source: Adapted from Pânico et al.^15^

High probability of DVT: 3 points or more; moderate probability: from 1 to 2 points; low probability: from 0 to -2 points.

To analyze whether investigations were conducted in an appropriate manner, it was considered appropriate to order a DD assay as initial investigation test if the WS score indicated low probability, in line with the recommendations contained in the 2015 SBACV DVT diagnosis and treatment guidelines. The DD assays were conducted using immunoturbidimetry. Results less than or equal to 500 ngFEU/mL (fibrinogen equivalent units) were considered normal. For patients over the age of 50 years, the cutoff for normality was found by multiplying the patient’s age by 10.^19^^,^^20^

Definitive diagnoses of DVT were confirmed using the CDUS reports available in patients’ medical records.

In turn, requests for CDUS examinations were considered justified when patients had a low WS probability of events but DD over the 500 ngFEU/mL cutoff point, or had moderate or high probability according to their WS.

Diagnostic strategies were considered inappropriate if: (1) DD was ordered as initial test in patients with moderate or high probability according to the WS, in whom its use is questionable; (2) CDUS was requested in patients with low probability according to the WS and a negative DD result; or (3) diagnosis was not supplemented with CDUS in patients with moderate or high pre-test probability or low pre-test probability and DD result over the cutoff point mentioned above.^9^^,^^14^ These rules were used to calculate the overall rate of inappropriate diagnostic management.

The study was approved by the institution’s Research Ethics Committee, consolidated opinion number 3.383.755.

### Statistical analysis

Data were tabulated and analyzed using SPSS 16.0. Continuous variables were expressed as measures of central tendency and dispersion, and categorical variables were expressed as frequencies and percentages. Descriptive analyses were performed with calculation of a 95% confidence interval (CI) for each point estimated.

## RESULTS

**Figure 1:**
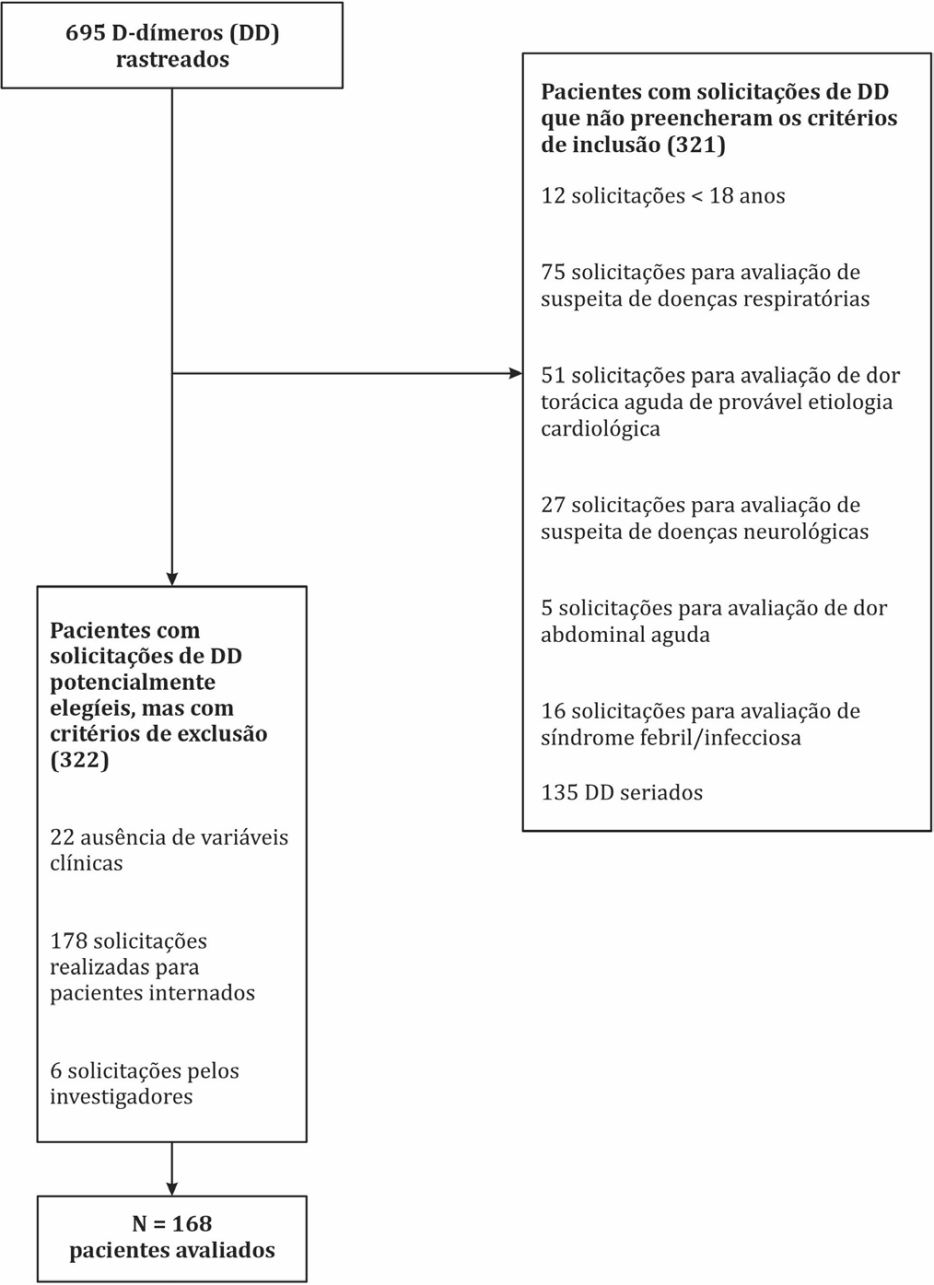
Flow diagram illustrating inclusion and exclusion of patients with suspected deep venous thrombosis, seen at a tertiary hospital in Santa Catarina, Brazil, from January to December 2018.

As illustrated in Figure 1, the final sample comprised 168 patients analyzed, 94 (55.9%) of whom were male.

The mean of age of the patients was 53 years, with a standard deviation of ± 17.6 years. The most prevalent signs and symptoms in the patients with suspected DVT were pain, in 125 patients (74.4%), and edema, in 118 patients (70.2%). Additional characteristics are listed in Table 2.

**Table 2:** Epidemiological and clinical profile of patients with suspected deep venous thrombosis (DVT) seen at a tertiary hospital in Santa Catarina, Brazil, from January to December 2018.

**Variable**	**N (168)**	**%**	**95%CI**
**Mean age**		53.3±17.6^†^	50-55
**Sex**			
Male	94	56.0	48-63
Female	74	44.0	36-63
**Ethnicity**			
White	149	88.7	83-93
Not white	19	11.3	10-11
**Signs and symptoms**			
Pain	126	75	68-81
Edema	118	70.2	63-77
Signs of inflammation^*^	24	14	9.0-23
Clubbing	12	7.1	3.0-11
Cyanosis	09	5.4	2.0-9.0
Paresthesias	07	4.2	3.0-4.3

* Heat, rubor, and erythema;

† Standard deviation.

95%CI: 95% confidence interval.

A total of 27 patients (16.1%) had a diagnosis of DVT confirmed by CDUS and the most common risk factor among patients with suspected DVT was age over 65 years, as shown in Tables 3 and 4 respectively.

**Table 3:** Diagnoses of patients with suspected deep venous thrombosis (DVT), seen at a tertiary hospital in Santa Catarina, Brazil, from January to December 2018.

**Variable**	**N (168)**	**%**	**95%CI**
DVT	27	16.1	15-31.7
Erysipelas	13	7.7	7.4-8.0
Thrombophlebitis	09	5.3	5.0-5.6
PAOD	08	4.7	4.5-5.0
Cellulitis	06	3.5	2.7-3.7
Chronic venous disease	04	2.3	2.2-2.5
Superficial venous thrombosis	03	1.7	1.6-3.4
Undefined diagnosis	84	50.5	49-50.5
Other diagnoses	14	8.3	8.0-8.6

PAOD: peripheral arterial occlusive disease; 95%CI: 95% confidence interval.

**Table 4:** Most common risk factors in patients with suspected deep venous thrombosis (DVT), seen at a tertiary hospital in Santa Catarina, Brazil, from January to December 2018.

**Risk factors**	**N (168)**	**%**	**95%CI**
Age > 65 years	43	25.5	25-26
Chronic venous disease	18	10.7	10-11
Prior history of DVT	12	7.1	6.0-7.4
Immobility	12	7.1	6.0-7.4
Prior orthopedic procedures	11	6.5	6.0-6.8
Family history of DVT	10	5.9	5.0-6.2
Obesity	7	4.2	3.0-4.3
Cancer	6	3.6	3.0-9.6
Trauma/fracture	5	3.0	2.2-3.1
Hormonal contraceptive method	4	2.4	2.2-2.5
Connective tissue diseases	4	2.4	2.2-2.5
Thrombophilias	1	0.6	0.5-0.6

95%CI: 95% confidence interval.

**Figure 2:**
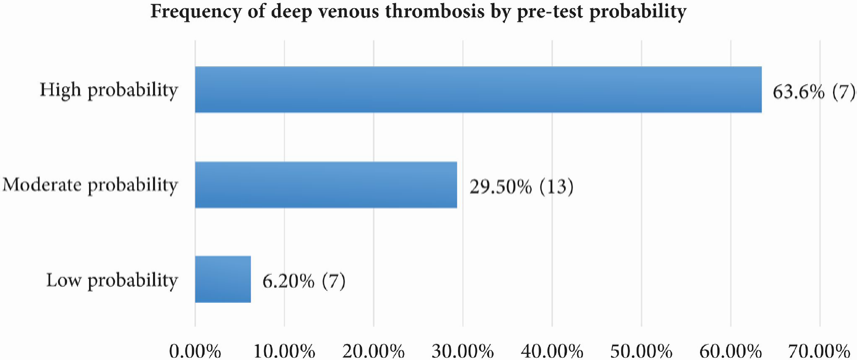
Frequency of deep venous thrombosis (DVT) by pre-test probability in patients with suspected DVT, seen at a tertiary hospital in Santa Catarina, Brazil, from January to December 2018.

**Figure 3:**
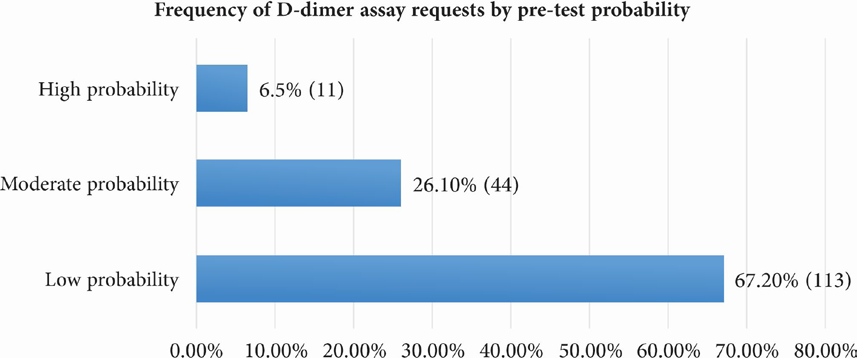
Frequency of D-dimer (DD) assay requests, by pre-test probability, in patients with suspected deep venous thrombosis, seen at a tertiary hospital in Santa Catarina, Brazil, from January to December 2018.

The prevalence of DVT increased significantly in line with pre-test DVT probability according to the WS (Figure 2). Figure 3 illustrates the frequency of requests for DD assays by pre-test probability.

**Figure 4:**
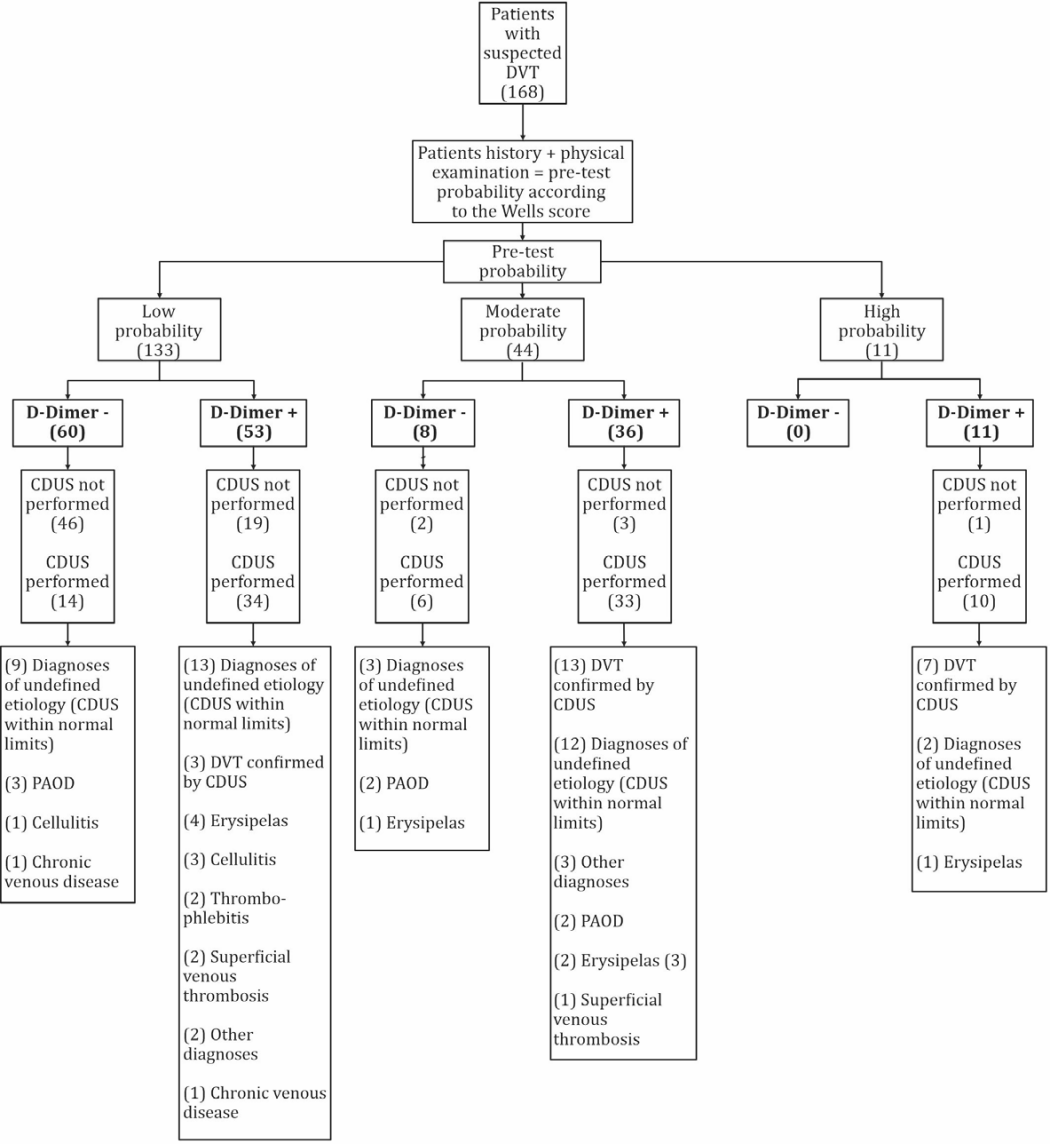
Flow diagram illustrating the diagnostic management adopted in patients with suspected deep venous thrombosis (DVT), seen at a tertiary hospital in Santa Catarina, Brazil, from January to December 2018. CDUS: color Doppler ultrasonography; PAOD: peripheral arterial occlusive disease.

It was found that DD was ordered for 55 patients (32.7%; 95%CI: 31-34) with suspected DVT, even though they had a moderate or high probability of DVT according to the WS. Moreover, in 14 cases (8.3%; 95%CI: 4.4-12.2) of patients with low probability CDUS was requested regardless of a DD result below the cutoff, while CDUS was not used to confirm or rule out the diagnosis in 19 patients (11.3%; 95%CI: 0.8-14.5) who had low probability according to the WS but elevated DD results. In just two cases (1.19%; 95%CI: 0.9-11.8), one with moderate and the other with high probability according to the WS, CDUS was not requested, as illustrated in Figure 4.

As such, CDUS was requested inappropriately in 35 cases (20.8%; 95%CI: 18.5-23). Overall, 90 cases (53.5%; 95%CI: 52.4-54.6) had inappropriate diagnostic management.

## DISCUSSION

In the present study, there was a higher prevalence of male patients, in contrast with the results of other studies in which the prevalence of female patients was higher.^21^^-^23 Epidemiological studies demonstrate that, although cases of VTE increase with age among both males and females, age adjusted rates are higher among men from 45 years of age onwards.^3^^,^^23^ This finding is confirmed by the mean age of the cases included in the present study, which was 53 years. With regard to the prevalence of risk factors, our data show that age over 65 years was the most prevalent risk factor. Deep vein thrombosis is rare among children, but its prevalence increases exponentially from the second to the eighth decades of life.^24^ In a study conducted by Kniffin et al., cases of DVT increased significantly from 65 years of age onwards: the incidence was 1.8 cases in every 1,000 from 65 to 69 years, increasing to 3.5 cases at 85 to 99 years of age.^25^ According to the 2015 SBACV DVT guidelines, the proportional increase in DVT cases with age suggests that this is the principal determinant factor in a first thrombotic event.^15^ The higher prevalence of other factors is also noteworthy, such as chronic venous insufficiency, trauma, and prior history of DVT.^25^

With regard to signs and symptoms, the literature confirms the findings of the present study, demonstrating that the complaints most frequently reported by patients with suspected and confirmed diagnosis of DVT are pain and asymmetrical edema of the lower limbs.^17^^,^^18^

A diagnosis of DVT was confirmed in 16.1% of cases, a similar rate to those observed in other studies that have assessed individuals with suspected DVT.^26^^,^^27^ As expected, the prevalence of confirmed DVT increased through higher strata of pre-test probability. A meta-analysis demonstrated that the frequency of DVT in groups with low, moderate, and high probability according to the WS were, respectively, 5.0% (95%CI: 4.0-8.0%), 17% (95%CI: 13-23%), and 53% (95%CI: 44-61%), which are similar results to those of the present study, particularly with regard to the variations in CI shown above.^9^

With respect to the primary objective of this study, it is important to be clear that the pre-test probability scores were designed and validated to increase diagnostic efficiency when investigating certain diseases. As such, Wells et al. demonstrated and validated use of the WS followed by DD testing for initial assessment of patients with suspected VTE.^9^ It is not clear whether the emergency department physicians know how to use this model of management correctly in their clinical practice.

In our study, in 32.7% of cases, DD tests were ordered when there was a moderate or high probability of DVT according to the WS. In comparison, a single center study found higher rates of DD requests in patients with a probable DVT diagnosis, with 52.7% of inappropriate test orders.^28^ In a different study with a similar objective, Arnason et al.^21^ observed 30% of inappropriate use of DD testing, which is a more similar rate to the one reported in the present study. Kristoffersen et al.^29^ conducted a multicenter study in Europe using questionnaires and demonstrated that 6% of the physicians surveyed ordered DD in inappropriate situations. In the past, all patients with suspected VTE would undergo imaging exams, but this approach was inefficient and expensive, since many patients with a suspicion did not have a diagnosis confirmed.^30^ Use of DD combined with a low probability WS can safely rule out VTE and reduce costs related to unnecessary requests for imaging exams. In this context, our study observed that in 8.3% of cases CDUS was used unnecessarily in patients with a low probability of diagnosis of DVT and a DD result below the cutoff point. As expected, these examinations did not confirm presence of DVT. However, false-positive results are more frequent when imaging exams are used in situations of low probability.^9^ Mousa et al. found excessively high rates (69%) of CDUS use in patients without indications for this examination. Use of this strategy is not indicated in situations of low probability of DVT and DD levels below the cutoff point.^31^ As demonstrated, in 19 cases (11.3%) there were failures to follow the diagnostic strategy for investigation of suspected DVT. Patients with a low probability WS, but an elevated DD result were not followed-up with CDUS, whereas among patients with moderate or high probability according to the WS, CDUS was not requested in just one case in each stratum.

A prospective study designed to validate diagnostic algorithms for pulmonary thromboembolism (PTE) observed that 92 of the 930 (9.9%) patients analyzed were not tested as recommended by the protocol. Of these, 5% exhibited PTE or DVT during the months of follow-up.^32^ This evidence supports the current data in showing that, in addition to excessive use of resources, under-testing can also be a problem in this context. With regard to the appropriateness of diagnostic strategies used to manage patients with suspected DVT, it was found that overall there was inappropriate management in more than half of the cases analyzed, whether because of inappropriate ordering of DD assays or because of incorrect indication of CDUS. A study published by Arnason et al.^21^ also observed that investigation of DVT in emergency was conducted incorrectly in about 25% of cases.

As such, the main utility of this study was to demonstrate inconsistencies in diagnostic investigation of patients with suspected DVT, suggesting that the recommendations set out in guidelines are not being correctly followed in clinical practice.

Cabana et al.^33^ conducted a study that assessed the causes of physicians’ low compliance with the guidelines, demonstrating that ignorance of guidelines was one of the most important causes of poor adherence to them.

The present study was not designed to conduct this type of assessment, but, as shown by the results observed, there is a clear need for better educational support to increase physicians’ knowledge about the current recommendations for management of DVT and to improve their understanding of the probability systems to support decision-making and rational use of supplementary examinations and tests.

A flow diagram illustrating selection for this study shows that DD assays were also ordered for patients with presentations related to etiologies other than DVT. This study therefore also reveals possible unnecessary use of this assay in other diagnostic scenarios, probably involving increased expenditure for the Unified Health System (SUS - Sistema Único de Saúde). Together with other available evidence, this underscores the need for further studies, preferably prospective national registries, to better analyze these issues related to diagnosis and management of VTE.

This study has some limitations. The absence of a clinical protocol for VTE management at the hospital studied makes it difficult to determine the reasons why DD assays were ordered and why CDUS was not requested for patients for whom it was indicated.

The low quality of medical records meant that it was not possible to reliably determine the diagnoses of many of the patients in whom DVT was probably ruled out. In an emergency setting, having ruled out the probability of DVT, the physicians chose non-specific diagnostic hypotheses such as pains and/or edema without definitive etiology, followed by discharge of the patient with guidance. Since follow-up was not possible, this study does not have the power to determine what happened afterwards. In view of this, we decided to adapt the research protocol and define these patients’ diagnosis as “diagnosis of undefined etiology”.

Moreover, because of the wide range of variation, it was necessary to employ the variable “other diagnoses” to group patients given diagnoses of other conditions that are part of the differential diagnosis of DVT or which could predispose to DVT, such as lymphedema, arthritis, ruptured Baker cyst, pain and edema related to orthopedic trauma (four cases), sickle cell crisis, decompensated liver disease, decompensated heart failure (two cases), and muscle distension (three cases).

The absence of a record of WS on the majority of patient records meant that the WS had to be calculated retrospectively, which could result in measurement bias, since the variable in the score that indicates a need to hypothesize “diagnoses more or less probable than DVT” is dependent on the investigator’s judgment. The sample was selected based on data on requests for DD assay and it is therefore possible that patients who were only investigated using imaging exams have been omitted from the sample.

Despite these limitations and despite the study having been conducted at a single center, affiliated to the SUS, the data reported here are similar to and replicated by other evidence available in the literature.

## CONCLUSIONS

There was a predominance of males among the patients with suspected DVT admitted to emergency. Pain and edema were the most frequent signs and symptoms among patients with a diagnostic suspicion of DVT.

The most common risk factor for DVT among these patients was age over 65 years. It was observed from the results obtained that there are divergences between clinical practice and the recommendations for diagnostic assessment of patients with suspected DVT, due to inappropriate use of diagnostic tests and examinations.

## References

[1] Tritschler T, Kraaijpoel N, Le Gal G, Wells PS (2018). Venous thromboembolism: Advances in Diagnosis and Treatment. JAMA.

[2] Anderson FA, Wheeler HB, Goldberg RJ (1991). A population-based perspective of the hospital incidence and case fatality rates of deep vein thrombosis and pulmonary embolism: the Worcester DVT study. Arch Intern Med.

[3] White RH (2003). The epidemiology of venous thromboembolism. Circulation.

[4] Barros MVL, Pereira VSR, Pinto DM (2012). Controvérsias no diagnóstico e tratamento da trombose venosa profunda pela ecografia vascular. J Vasc Bras.

[5] Segal JB, Eng J, Tamariz LJ, Bass EB (2007). Review of the evidence on diagnosis of deep venous thrombosis and pulmonary embolism. Ann Fam Med.

[6] Wells PS, Anderson DR, Bormanis J (1997). Value of assessment of pretest probability of deep-vein thrombosis in clinical management. Lancet.

[7] Bounameaux H, Cirafici P, De Moerloose P (1991). Measurement of D-dimer in plasma as diagnostic aid in suspected pulmonary embolism. Lancet.

[8] Huisman MV, Klok FA (2013). Diagnostic management of acute deep vein thrombosis and pulmonary embolism. J Thromb Haemost.

[9] Wells PS, Owen C, Doucette S, Fergusson D, Tran H (2006). Does this patient have deep vein thrombosis?. JAMA.

[10] Gaitini D (2006). Current approaches and controversial issues in the diagnosis of deep vein thrombosis via duplex doppler ultrasound. J Clin Ultrasound.

[11] Choosing Wisely Initiative ABIM foundation.

[12] Fortes VB, Rollo HA, Fortes AT (2007). Avaliação do modelo de predição clínica de Wells et al. no diagnóstico da trombose venosa profunda dos membros inferiores. J Vasc Bras.

[13] Rollo HA, Fortes VB, Fortes AT (2005). Abordagem diagnóstica dos pacientes com suspeita de trombose venosa profunda dos membros inferiores. J Vasc Bras.

[14] Thachil J, Fitzmaurice DA, Toh CH (2010). Appropriate use of d-dimer in hospital patients. Am J Med.

[15] Pânico MDB, Matielo MF, Porto CLL, Marques MA, Yoshida RDA (2015). Projeto Diretrizes SBACV. Trombose venosa profunda diagnóstico e tratamento..

[16] Dean AG, Sullivan KM, Soe MM OpenEpi: Open Source Epidemiologic Statistics for Public Health. Versão 3.01.

[17] Teismann NA, Cheung PT, Frazee B (2009). Is the ordering of imaging for suspected venous thromboembolism consistent with D- dimer result?. Ann Emerg Med.

[18] Wells PS, Anderson DR, Rodger M (2003). Evaluation of D-dimer in the diagnosis of suspected deep-vein thrombosis. N Engl J Med.

[19] Stein PD, Hull RD, Patel KC (2004). D-Dimer for the exclusion of acute venous thrombosis and pulmonary embolism. A systematic review. Ann Intern Med.

[20] Righini M, Van Es J, Den Exter PL (2014). Age-adjusted d-dimer cutoff levels to rule out pulmonary embolism: the ADJUST-PE study. JAMA.

[21] Arnason T, Wells PS, Forster AJ (2007). Appropriateness of diagnostic strategies for evaluating suspected venous thromboembolism. Thromb Haemost.

[22] Courtney DM, Kline JA, Kabrhel C (2010). Clinical features from the history and physical examination that predict the presence or absence of pulmonary embolism in symptomatic emergency department patients: Results of a prospective, multicenter study. Ann Emerg Med.

[23] Heit JA, Spencer FA, White RH (2016). The epidemiology of venous thromboembolism. J Thromb Thrombolysis.

[24] Bulger CM, Jacobs C, Patel NH (2004). Epidemiology of acute deep vein thrombosis. Tech Vasc Interv Radiol.

[25] Kniffin WD, Baron JA, Barrett J, Birkmeyer JD, Anderson FA (1994). The epidemiology of diagnosed pulmonary embolism and deep venous thrombosis in the Eldery. Arch Intern Med.

[26] Shields GP, Turnipseed S, Panacek EA, Melnikoff N, Gosselin R, White RH (2002). Validation of the Canadian clinical probability model for acute venous thrombosis. Acad Emerg Med.

[27] Kearon C, Ginsberg GS, Douketis JD (2001). Management of suspect deep venous thrombosis in outpatients by using clinical assessment and D-dimer testing. Ann Intern Med.

[28] Kamolratanapiboon K, Tantanate C (2019). Inappropriate use of D-dimer and impact on the test characteristics for deep vein thrombosis exclusion. Scand J Clin Lab Invest.

[29] Kristoffersen AH, Ajzner E, Rogic D (2016). Is d-dimer used according to clinical algorithms in the diagnostic work-up patients with suspicion of venous thromboembolism? A study in six European countries. Thromb Res.

[30] Wells PS, Anderson DR (2000). Diagnosis of deep-vein thrombosis in the year 2000. Curr Opin Pulm Med.

[31] Mousa AY, Broce M, Gill G, Kali M, Yacoub M, AbuRahma AF (2015). Appropriate use of D-dimer testing can minimize over-utilization of venous duplex ultrasound in a contemporary high-volume hospital. Ann Vasc Surg.

[32] Wells PS, Anderson DR, Rodger M (2001). Excluding pulmonary embolism at the bedside without diagnostic imaging: management of patients with suspected pulmonary embolism presenting to the emergency department by using a simple clinical model and d-dimer. Ann Intern Med.

[33] Cabana MD, Rand CS, Powe NR (1999). Why don’t physicians follow clinical practice guidelines? A framework for improvement. JAMA.

