# Supplementary material for: Evaluation of the diagnostic management of deep vein thrombosis in the emergency department of a tertiary hospital in Santa Catarina, Brazil: a cross-sectional study
Source: J Vasc Bras. 2022 Oct 3;21:e20200217. doi: 10.1590/1677-5449.202002171 (PMC9543468; doi:10.1590/1677-5449.202002171)
Supplement: Translation

# Translation: Avaliação do manejo diagnóstico de trombose venosa profunda no departamento de emergência de um hospital terciário em Santa Catarina: um estudo transversal

**DOI:** 10.1590/1677-5449.202002172

**Authors:** Lucas Tramujas, Márcio Mesquita Judice, Angela Bueno Becker

**Affiliations:** 1 Hospital Governador Celso Ramos, Florianópolis, SC, Brasil.; 2 Instituto de Pesquisa Hcor, São Paulo, SP, Brasil.

**Keywords:** trombose venosa, diagnóstico, protocolos clínicos

## Abstract

**Contexto:**

O tromboembolismo venoso é uma entidade que compreende a trombose venosa profunda e o tromboembolismo pulmonar. Embora os protocolos para diagnóstico dessas doenças estejam bem definidos, evidências têm demonstrado uso inadequado de recursos diagnósticos.

**Objetivos:**

Definir o perfil epidemiológico dos pacientes com suspeita de trombose venosa profunda admitidos na emergência, determinar taxas de inadequação nas solicitações de D-dímero e eco-Doppler colorido venoso de membros inferiores e identificar se essas solicitações seguiram as recomendações da diretriz da Sociedade Brasileira de Angiologia e Cirurgia Vascular de 2015.

**Métodos:**

Estudo observacional transversal que avaliou retrospectivamente 168 pacientes com suspeita de trombose venosa profunda, aos quais foi solicitado D-dímero. Foram mensurados os fatores de risco mais comuns e a probabilidade pré-teste pelo escore de Wells. O perfil epidemiológico desses pacientes, assim como as taxas de inadequação, foram avaliados por meio de uso de estatística descritiva.

**Resultados:**

Em 55 (32,7%) casos, as solicitações de D-dímero foram inadequadas. Em 14 (8,3%) pacientes com baixa probabilidade no escore de Wells e D-dímero negativo, houve uso desnecessário de eco-Doppler colorido venoso de membros inferiores, sendo que, em 19 (11,3%) daqueles com baixa probabilidade no escore de Wells e D-dímero elevado, não houve complementação diagnóstica. O uso de eco-Doppler colorido venoso foi inadequado em 35 (20,8%) casos. A taxa global de inadequação foi de 53,5%.

**Conclusões:**

Constataram-se divergências entre a prática clínica e as recomendações propostas para avaliação diagnóstica nos pacientes com suspeita de trombose venosa profunda devido ao uso inadequado de testes diagnósticos.

## INTRODUÇÃO

Dados provenientes de registros americanos estimam que a incidência de tromboembolismo venoso (TEV) tem se mantido constante ao longo do tempo, com uma taxa de 0,7 a 1,4 casos por 1.000 pessoas-ano^1^. Estima-se que cerca de dois terços desses sejam representados por casos de trombose venosa profunda (TVP)^2^^,^^3^.

O diagnóstico clínico da TVP é um desafio, visto que os sinais e sintomas clássicos nem sempre são observados. Assim, a associação dos achados clínicos com fatores de risco agrupados a um sistema de predição e uso de exames complementares constitui a melhor forma de realização do diagnóstico^4^.

Dessa forma, o uso de D-dímero (DD), um produto da degradação da fibrina originado durante a ocorrência de eventos trombóticos, e do eco-Doppler colorido (EDC) venoso de membros inferiores assume papel central na investigação de casos suspeitos de TVP^5^^,^^6^.

Os níveis séricos de DD aumentam significativamente em uma série de doenças, com a idade e em situações fisiológicas como a gravidez, sendo, portanto, um marcador de alta sensibilidade e baixa especificidade, com alto valor preditivo negativo para TEV^7^. Portanto, a principal recomendação é que ele seja utilizado como exame inicial de investigação em situações de baixa probabilidade no pré-teste para o diagnóstico de TVP, em que a acurácia, em casos de exame negativo, é suficiente para excluir a possibilidade de doença^8^^,^^9^.

Todavia, o diagnóstico por imagem é necessário para o diagnóstico de TVP. Nessa situação, o EDC é o método de escolha para diagnóstico, com sensibilidade de 96% e especificidade superior a 98%, a depender da região examinada^10^.

No ano de 2012, nos Estados Unidos (EUA), o American Board of Internal Medicine (ABIM) lançou uma campanha denominada Choosing Wisely International. Essa iniciativa vai de encontro ao paradigma do “menos é mais”, orientada a não realização de condutas e testes desnecessários^11^.

Estudos recentes têm apontado para o uso inadequado de recursos diagnósticos na avaliação de TEV, com destaques para alguns poucos estudos nacionais que avaliaram a questão^12^^-^14.

Nesse sentido, esta pesquisa pretende gerar mais dados a respeito do manejo diagnóstico da TVP, apontando para possíveis incongruências na avaliação desses pacientes e dando ênfase de forma indireta para o possível impacto econômico no uso inadequado dos recursos diagnósticos.

Dessa forma, os principais objetivos deste estudo foram avaliar o perfil clínico e epidemiológico dos pacientes com suspeita de TVP, verificar a taxa de inadequação na solicitação de DD e EDC e definir se a investigação de TVP esteve de acordo com as recomendações propostas pela diretriz de diagnóstico e tratamento de TVP da Sociedade Brasileira de Angiologia e Cirurgia Vascular (SBACV) de 2015 em um hospital terciário em Santa Catarina^15^.

## MÉTODOS

Tratou-se de um estudo observacional transversal que avaliou as solicitações de DD em pacientes com suspeita de TVP na emergência do Hospital Governador Celso Ramos (Florianópolis, estado de Santa Catarina), de janeiro a dezembro de 2018. Foi utilizado o *software* Open Epi para cálculo amostral^16^. O tamanho da amostra estimado foi de 139 indivíduos, considerando-se uma estimativa arbitrária de frequência de inadequação global de 10% devido à ampla variação da taxa de inadequação apresentada na literatura em pacientes com suspeita de TVP^17^. A precisão absoluta foi de 5%, e o nível de significância adotado foi de 5%.

As variáveis foram coletadas por meio de protocolo de pesquisa elaborado pelos pesquisadores.

A partir dos dados fornecidos pelo laboratório da instituição, identificaram-se todos os pacientes para os quais foram solicitados DD; posteriormente, os prontuários foram analisados.

Foram definidos como critérios de inclusão os seguintes: (1) idade maior ou igual a 18 anos; (2) pacientes com sinais e sintomas sugestivos de TVP, como dor, edema, cianose, empastamento de panturrilhas, dilatação do sistema venoso superficial ou alterações compatíveis no membro acometido durante o exame físico; e (3) inclusão de apenas um exame de DD por paciente avaliado na emergência.

Os critérios de exclusão determinados foram: (1) ausência de variáveis clínicas necessárias para o cálculo da probabilidade através do escore de Wells (EW); (2) suspeita de TVP em pacientes internados; e (3) pacientes que foram avaliados pelos investigadores da pesquisa durante trabalho assistencial.

Mensurou-se o registro dos principais fatores de risco para TVP, que foram: idade maior que 65 anos, obesidade (índice de massa corporal [IMC] maior ou igual a 30 kg/m^2^), diagnóstico de neoplasia nos últimos 6 meses, doença venosa crônica, TVP prévia, procedimentos ortopédicos como artroplastia de quadril ou joelho ou artroscopia de joelho, imobilidade definida pela supressão dos movimentos articulares, seja por causas neurológicas, musculoesqueléticas ou devido a procedimento cirúrgico, trauma no último mês, uso de contraceptivos orais ou terapia de reposição hormonal, gravidez, puerpério, trombofilias genéticas ou hereditárias e história familiar de TEV^15^.

O EW modificado para TVP foi a ferramenta utilizada para o cálculo da probabilidade pré-teste, que estima em alta probabilidade para TVP um escore de 3 ou mais pontos, probabilidade moderada, de 1 a 2 pontos e baixa probabilidade, de 0 a -2 pontos (Tabela 1)^6^^,^^15^^,^^18^.

**Tabela 1:** Escore de Wells modificado para trombose venosa profunda (TVP), utilizado para avaliação de pacientes com suspeita de TVP atendidos em um hospital terciário em Santa Catarina, no período de janeiro a dezembro de 2018.

**Achado clínico**	**Pontos**
Câncer ativo (paciente recebeu previamente tratamento para câncer pelo menos há 6 meses ou está atualmente recebendo tratamento paliativo)	1
Paralisia, paresia ou imobilização de extremidade inferior	1
Imobilidade no leito por 3 dias ou mais ou cirurgia maior até 4 semanas	1
Aumento da sensibilidade ao longo das veias do sistema venoso profundo	1
Edema em todo membro	1
Edema da panturrilha > 3 cm em relação ao lado assintomático (medida realizada 10 cm abaixo da tuberosidade da tíbia)	1
Edema depressível (cacifo) maior na perna afetada (unilateral)	1
Veias colaterais superficiais	1
TVP prévia documentada	1
Diagnóstico alternativo mais provável que TVP	-2

Fonte: Adaptado de Pânico et al.^15^.

Alta probabilidade para TVP: 3 ou mais pontos; probabilidade moderada: de 1 a 2 pontos; baixa probabilidade: de 0 a -2 pontos.

Para analisar se a investigação foi realizada de forma apropriada, foram consideradas adequadas as solicitações de DD diante de EW de baixa probabilidade como exame inicial de investigação, conforme a recomendação proposta pela diretriz de diagnóstico e tratamento de TVP da SBACV de 2015. A dosagem de DD foi efetuada pelo método imunoturbidimétrico. Foram considerados normais resultados iguais ou inferiores a 500 ngFEU/mL (unidades equivalentes de fibrinogênio). Em pacientes acima dos 50 anos de idade, o valor limite para a normalidade foi encontrado multiplicando a idade por 10^19^^,^^20^.

Os diagnósticos definitivos de TVP foram confirmados por meio de laudo de EDC disponível em prontuário.

Quanto às solicitações de EDC, foram consideradas corretas as indicações em pacientes com baixa probabilidade de eventos e DD acima do ponto de corte de 500 ngFEU/mL ou em contexto de moderada ou alta probabilidade no EW.

Foram consideradas inadequadas as estratégias diagnósticas em que houve: (1) solicitação de DD como exame inicial em situação de moderada ou alta probabilidade no EW, em que seu uso é questionável; (2) uso de EDC em situação de baixa probabilidade no EW e DD negativo; (3) não complementação diagnóstica com EDC em pacientes com probabilidade pré-teste moderada ou alta ou probabilidade pré-teste baixa e DD acima do ponto de corte mencionado^9^^,^^14^. A partir disso, calculou-se a taxa global de inadequação.

O estudo foi aprovado pelo Comitê de Ética e Pesquisa da instituição, sob parecer consubstanciado número 3.383.755.

### Análise estatística

A análise e tabulação de dados foi realizada por meio do programa SPSS 16.0. As variáveis contínuas foram descritas como medidas de tendência central e dispersão, e as variáveis categóricas foram apresentadas como frequências e porcentagens. A análise descritiva foi realizada calculando-se o intervalo de confiança (IC) de 95% para cada ponto estimado.

## RESULTADOS

Conforme demonstrado na Figura 1, a amostra final foi composta por 168 pacientes avaliados, sendo 94 (55,9%) do sexo masculino.

**Figura 1:** Fluxograma de inclusões e exclusões em pacientes com suspeita de trombose venosa profunda, atendidos em um hospital terciário em Santa Catarina, no período de janeiro a dezembro de 2018.

A média de idade dos pacientes foi de 53 anos, com desvio-padrão de ± 17,6 anos. Os sintomas e sinais mais prevalentes em pacientes com suspeita de TVP foram dor em 125 pacientes (74,4%) e edema em 118 pacientes (70,2%). As demais características estão descritas na Tabela 2.

**Tabela 2:** Perfil epidemiológico e clínico de pacientes com suspeita de trombose venosa profunda (TVP) atendidos em um hospital terciário em Santa Catarina, no período de janeiro a dezembro de 2018.

**Variável**	**N (168)**	**%**	**IC95%**
**Idade média**		53,3±17,6^†^	50-55
**Sexo**			
Masculino	94	56,0	48-63
Feminino	74	44,0	36-63
**Etnia**			
Brancos	149	88,7	83-93
Não brancos	19	11,3	10-11
**Sinais e sintomas**			
Dor	126	75	68-81
Edema	118	70,2	63-77
Sinais flogísticos*	24	14	9,0-23
Empastamento	12	7,1	3,0-11
Cianose	09	5,4	2,0-9,0
Parestesias	07	4,2	3,0-4,3

* Calor, rubor e eritema;

† Desvio-padrão.

IC95%: intervalo de confiança de 95%.

O diagnóstico de TVP confirmado por EDC foi realizado em 27 pacientes (16,1%), sendo que o fator de risco mais evidenciado nos pacientes com suspeita de TVP foi idade acima de 65 anos, conforme disposto respectivamente nas Tabelas 3 e 4.

**Tabela 3:** Diagnósticos realizados em pacientes com suspeita de trombose venosa profunda (TVP), atendidos em um hospital terciário em Santa Catarina, no período de janeiro a dezembro de 2018.

**Variável**	**N (168)**	**%**	**IC95%**
TVP	27	16,1	15-31,7
Erisipela	13	7,7	7,4-8,0
Tromboflebite	09	5,3	5,0-5,6
DAOP	08	4,7	4,5-5,0
Celulite	06	3,5	2,7-3,7
Doença venosa crônica	04	2,3	2,2-2,5
Trombose venosa superficial	03	1,7	1,6-3,4
Diagnósticos não definidos	84	50,5	49-50,5
Outros diagnósticos	14	8,3	8,0-8,6

DAOP: doença arterial obstrutiva periférica; IC95%: intervalo de confiança de 95%.

**Tabela 4:** Fatores de risco mais comuns em pacientes com suspeita de trombose venosa profunda (TVP), atendidos em um hospital terciário em Santa Catarina, no período de janeiro a dezembro de 2018

**Fatores de risco**	**N (168)**	**%**	**IC95%**
Idade > 65 anos	43	25,5	25-26
Doença venosa crônica	18	10,7	10-11
História de TVP prévia	12	7,1	6,0-7,4
Imobilidade	12	7,1	6,0-7,4
Procedimentos ortopédicos prévios	11	6,5	6,0-6,8
História familiar de TVP	10	5,9	5,0-6,2
Obesidade	7	4,2	3,0-4,3
Neoplasia	6	3,6	3,0-9,6
Trauma/fratura	5	3,0	2,2-3,1
Método contraceptivo hormonal	4	2,4	2,2-2,5
Colagenoses	4	2,4	2,2-2,5
Trombofilias	1	0,6	0.5-0.6

IC95%: intervalo de confiança de 95%.

A prevalência de TVP aumentou significativamente conforme a probabilidade no EW (Figura 2). Na Figura 3, estão dispostas as frequências de solicitações de DD, conforme probabilidade pré-teste.

**Figura 2:** Frequência de trombose venosa profunda (TVP) conforme probabilidade pré-teste em pacientes com suspeita de TVP, atendidos em um hospital terciário em Santa Catarina, no período de janeiro a dezembro de 2018.

**Figura 3:** Frequência de solicitações de D-dímero (DD), conforme probabilidade pré-teste em pacientes com suspeita de trombose venosa profunda, atendidos em um hospital terciário em Santa Catarina, no período de janeiro a dezembro de 2018.

Constatou-se que, em 55 dos pacientes (32,7%; IC95%: 31-34) com suspeita de TVP, a solicitação de DD foi realizada, apesar da moderada ou alta probabilidade no EW. Nos indivíduos com baixa probabilidade e DD abaixo do ponto do corte, houve solicitação de EDC em 14 casos (8,3%; IC95%: 4,4-12,2), enquanto em 19 pacientes (11,3%; IC95%: 0,8-14,5) que se apresentaram com baixa probabilidade no EW e DD elevado, não houve complementação com EDC para definição diagnóstica. Em apenas dois casos (1,19%; IC95%: 0,9-11,8), um de moderada e outro de alta probabilidade no EW, não houve complementação com EDC, conforme demonstrado na Figura 4.

**Figura 4:** Fluxograma demonstrando o manejo diagnóstico adotado em pacientes com suspeita de trombose venosa profunda (TVP), atendidos em um hospital terciário em Santa Catarina, no período de janeiro a dezembro de 2018. EDC: eco-Doppler colorido; DAOP: doença arterial obstrutiva periférica.

O uso de EDC foi inadequado em 35 casos (20,8%; IC95%: 18,5-23). A taxa global de inadequação foi de 90 casos (53,5%; IC95%: 52,4-54,6).

## DISCUSSÃO

No presente estudo, verificou-se uma maior prevalência de indivíduos do sexo masculino, divergentes de outros em que houve maior prevalência de pacientes do sexo feminino^21^^-^23. Estudos epidemiológicos demonstram que, embora os casos de TEV aumentem com a idade tanto no sexo masculino quanto feminino, as taxas ajustadas para idade passam a ser maiores em homens, principalmente a partir dos 45 anos^3^^,^^23^. Esse dado pode ser reafirmado pela média de idade dos casos incluídos na presente pesquisa, que foi de 53 anos. Quanto à prevalência dos fatores de risco, nossos dados demonstram que a idade acima de 65 anos foi o fator de risco mais prevalente. A TVP é rara em crianças, mas sua prevalência aumenta exponencialmente entre a segunda e oitava décadas de vida^24^. Em trabalho realizado por Kniffin et al., os casos de TVP aumentaram significativamente a partir dos 65 anos: a incidência foi de 1,8 casos para cada 1.000 entre os 65 e 69 anos, com um aumento de 3,5 casos entre os 85 e 99 anos de idade^25^. Não obstante, segundo a diretriz de TVP da SBACV de 2015, o aumento proporcional de casos de TVP com a idade sugere que esse seja o principal fator determinante para um primeiro evento trombótico^15^. Destaca-se também maior prevalência de outros fatores, como insuficiência venosa crônica, trauma e história prévia de TVP^25^.

Quanto aos sinais e sintomas, a literatura confirma os presentes achados ao demonstrar que as queixas mais frequentemente relatadas em pacientes com suspeita diagnóstica e TVP confirmados são dor e edema assimétricos de membros inferiores^17^^,^^18^.

O diagnóstico de TVP foi realizado em 16,1% dos casos, taxa semelhante à encontrada em estudos que avaliaram indivíduos com suspeita de TVP^26^^,^^27^. Como esperado, a prevalência de TVP aumentou conforme os estratos de probabilidade no pré-teste. Uma metanálise demonstrou que, nos grupos de baixa, moderada e alta probabilidade no EW, respectivamente, as frequências variaram em 5,0% (IC95%: 4,0-8,0%), 17% (IC95%: 13-23%), 53% (IC95%: 44-61%), resultados similares ao do presente estudo, principalmente se considerarmos as variações dos IC acima demonstrados^9^.

Quanto ao principal objetivo desta pesquisa, é importante mencionar que os escores de probabilidade pré-teste foram desenhados e validados para aumentar a eficiência diagnóstica na avaliação de determinadas doenças. Nessa perspectiva, Wells et al. demonstraram e validaram o EW seguido do uso de DD para avaliação inicial de pacientes com suspeita de TEV^9^. Há dúvidas se os emergencistas sabem fazer uso correto desse modelo de abordagem na prática clínica.

Em nosso estudo, em 32,7% dos casos, as solicitações de DD foram realizadas diante de TVP com moderada ou alta probabilidade no EW. Em comparação, um estudo unicêntrico constatou taxas mais altas de solicitações em pacientes com provável diagnóstico dessa doença, com 52,7% de pedidos inadequados^28^. Arnason et al., em outro trabalho com objetivo semelhante, observaram 30% de inadequação ao uso de DD, taxas mais próximas das reportadas na presente pesquisa^21^. Kristoffersen et al.^29^, em estudo multicêntrico europeu baseado em questionários, demonstraram que 6% dos médicos solicitariam DD em situações inadequadas. No passado, todos os pacientes com suspeita de TEV eram submetidos a testes de imagem; entretanto, essa conduta era pouco eficiente e dispendiosa, pois muitos pacientes com suspeita não confirmavam o diagnóstico^30^. O uso de DD associado a um EW de baixa probabilidade possibilitou excluir TEV com segurança e reduzir custos decorrentes da solicitação inadequada de exames de imagem. Dentro desse contexto, nosso estudo observou que, em 8,3% dos casos, houve uso desnecessário de EDC em pacientes com baixa probabilidade de diagnóstico de TVP com DD abaixo do ponto de corte. Como esperado, os resultados desses exames não confirmaram a doença. Não obstante, resultados falso-positivos ocorrem mais frequentemente quando se faz uso de exames de imagem em situações de baixa probabilidade^9^. Mousa et al.^31^, em sua casuística, constataram taxas excessivamente altas (69%) de uso de EDC em pacientes sem indicação de serem submetidos ao exame. O uso dessa estratégia em situações de baixa probabilidade e DD abaixo do ponto de corte não é indicado. Como demonstrado, em 19 casos (11,3%), houve falha no seguimento da estratégia diagnóstica para avaliação de suspeita de TVP. Os pacientes com EW de baixa probabilidade, mas DD elevado não foram seguidos com EDC, além de que, nos pacientes com moderada ou alta probabilidade no EW, em apenas um caso em cada estrato de probabilidade não foi solicitado EDC.

Em um estudo prospectivo delineado para validar algoritmos diagnósticos para tromboembolismo pulmonar (TEP), 92 dos 930 (9,9%) pacientes avaliados não foram testados conforme sugerido pelo protocolo. Desses, nos meses de seguimento, 5% apresentaram TEP ou TVP^32^. Essa evidência ratifica esses dados ao determinar que, além do uso excessivo de recursos, nesse contexto a subtestagem também é um problema. Em relação à adequação das estratégias diagnósticas na abordagem de pacientes com suspeita de TVP, observou-se uma taxa de inadequação global em mais da metade dos casos analisados, quer seja pela solicitação inadequada de DD ou pela incorreta indicação do uso de EDC. No estudo publicado por Arnason et al., também foi observado que a investigação de TVP na emergência foi inapropriada em cerca de 25% dos casos^21^.

Sendo assim, a principal utilidade desta pesquisa foi demonstrar haver incongruências na investigação diagnóstica dos pacientes com suspeita de TVP, sugerindo que, na prática clínica, as recomendações propostas pelas diretrizes não são adequadamente seguidas.

Cabana et al.^33^, em estudo que avaliou as causas relacionadas à baixa aplicabilidade de médicos às diretrizes, demonstraram que o desconhecimento em relação às diretrizes foi uma das principais causas de baixa aderência.

A presente casuística não objetivou fazer esse tipo de avaliação, porém, de acordo com os resultados observados, fica clara a necessidade de maior suporte educacional para ampliar o conhecimento dos médicos sobre as recomendações vigentes no manejo da TVP, bem como maior entendimento dos sistemas de probabilidade para tomada de decisão e uso racional de exames complementares.

Conforme fluxograma de triagem do estudo, constatamos que houve solicitação de DD em pacientes com alterações relacionadas a outras etiologias que não TVP. Portanto, o presente estudo também apontou para um possível uso desnecessário desse exame em outros cenários diagnósticos, com provável aumento de custos para o Sistema Único de Saúde (SUS). Isso reforça, junto de outras evidências disponíveis, a necessidade de mais estudos, de preferência registros prospectivos nacionais, para melhor avaliar essas questões referentes ao diagnóstico e manejo do TEV.

Este estudo apresentou algumas limitações. A ausência de um protocolo clínico de abordagem para TEV no hospital avaliado dificultou a percepção do motivo por que os DD foram solicitados, bem como a compreensão da ausência da solicitação de EDC nos pacientes com indicação.

A baixa qualidade dos prontuários não permitiu avaliar com fidelidade o diagnóstico de muitos pacientes em que a TVP foi provavelmente excluída. Em um cenário de emergência, descartado a probabilidade de TVP, os médicos optaram por hipóteses diagnósticas pouco especificas como dores e/ou edema sem etiologia definida, com a posterior alta e orientação do paciente. Devido à impossibilidade de realizar o seguimento, a presente pesquisa não teve poder para definir o que ocorreu posteriormente. Em vista disso, optamos por adaptar o protocolo e definir o diagnóstico desses pacientes como “diagnósticos de etiologia não definida”.

Além de que, por sua ampla variedade, foi necessário utilizar a variável “outros diagnósticos” para agrupar pacientes que receberam diagnóstico de outras condições que fazem diagnóstico diferencial ou que podem predispor TVP, como linfedema, artrite, cisto de Baker roto, dor e edema relacionados a trauma ortopédico (quatro casos), crise falciforme, hepatopatia descompensada, insuficiência cardíaca descompensada (dois casos) e distensão muscular (três casos).

A ausência do registro do EW na maioria dos prontuários fez com que fosse necessário o seu cálculo de forma retrospectiva, o que pode resultar em viés de aferição, pois a variável que indica a necessidade de hipotetizar os “diagnósticos mais ou menos prováveis que TVP” no respectivo escore depende da interpretação do investigador. Essa amostra foi baseada nos dados de solicitação de DD; devido a isso, é possível que pacientes que tenham sido avaliados apenas com exame de imagem não tenham sido incluídos nessa casuística.

A despeito disso e de o estudo ter sido realizado em um único centro, vinculado ao SUS, os dados aqui mencionados foram semelhantes e replicados por outras evidências disponíveis na literatura.

## CONCLUSÕES

Nos pacientes com suspeita de TVP admitidos à emergência, houve uma maior prevalência de pacientes do sexo masculino. Dor e edema foram os sintomas e sinais mais frequentes nos pacientes com suspeita diagnóstica de TVP.

O fator de risco para TVP mais evidenciado nesses pacientes foi a idade acima de 65 anos. Constatou-se, por meio dos resultados obtidos, haver divergências entre a prática clínica e as recomendações propostas para avaliação diagnóstica dos pacientes com suspeita de TVP, devido ao uso inadequado de testes diagnósticos.
